# Potential Relevance of α_1_-Adrenergic Receptor Autoantibodies in Refractory Hypertension

**DOI:** 10.1371/journal.pone.0003742

**Published:** 2008-11-17

**Authors:** Katrin Wenzel, Hannelore Haase, Gerd Wallukat, Wolfgang Derer, Sabine Bartel, Volker Homuth, Florian Herse, Norbert Hubner, Herbert Schulz, Marion Janczikowski, Carsten Lindschau, Christoph Schroeder, Stefan Verlohren, Ingo Morano, Dominik N. Muller, Friedrich C. Luft, Rainer Dietz, Ralf Dechend, Peter Karczewski

**Affiliations:** 1 Medical Faculty of the Charité, Franz-Volhard Clinic and HELIOS Klinikum-Berlin, Experimental and Clinical Research Center, Berlin, Germany; 2 Max Delbrück Center for Molecular Medicine, Berlin, Germany; 3 Department of Nephrology, Hannover Medical School, Hannover, Germany; 4 E. R. D. E. eV, Berlin, Germany; University of Arizona, United States of America

## Abstract

**Background:**

Agonistic autoantibodies directed at the α_1_-adrenergic receptor (α_1_-AAB) have been described in patients with hypertension. We implied earlier that α_1_-AAB might have a mechanistic role and could represent a therapeutic target.

**Methodology/Principal Findings:**

To pursue the issue, we performed clinical and basic studies. We observed that 41 of 81 patients with refractory hypertension had α_1_-AAB; after immunoadsorption blood pressure was significantly reduced in these patients. Rabbits were immunized to generate α_1_-adrenergic receptor antibodies (α_1_-AB). Patient α_1_-AAB and rabbit α_1_-AB were purified using affinity chromatography and characterized both by epitope mapping and surface plasmon resonance measurements. Neonatal rat cardiomyocytes, rat vascular smooth muscle cells (VSMC), and Chinese hamster ovary cells transfected with the human α_1A_-adrenergic receptor were incubated with patient α_1_-AAB and rabbit α_1_-AB and the activation of signal transduction pathways was investigated by Western blot, confocal laser scanning microscopy, and gene expression. We found that phospholipase A2 group IIA (*PLA2-IIA*) and L-type calcium channel (*Cacna1c*) genes were upregulated in cardiomyocytes and VSMC after stimulation with both purified antibodies. We showed that patient α_1_-AAB and rabbit α_1_-AB result in protein kinase C alpha activation and transient extracellular-related kinase (EKR1/2) phosphorylation. Finally, we showed that the antibodies exert acute effects on intracellular Ca^2+^ in cardiomyocytes and induce mesentery artery segment contraction.

**Conclusions/Significance:**

Patient α_1_-AAB and rabbit α_1_-AB can induce signaling pathways important for hypertension and cardiac remodeling. Our data provide evidence for a potential clinical relevance for α_1_-AAB in hypertensive patients, and the notion of immunity as a possible cause of hypertension.

## Introduction

Autoimmunity with agonistic autoantibodies directed at endogenous receptors can cause Graves' disease through the thyrotropin receptor and promotes insulin release via CD38 [1], [2]. Agonistic antibodies that stimulate the angiotensin (Ang) II AT1 receptor have been described in preeclampsia and humorally mediated kidney transplant rejection [3], [4]. The most compelling case for agonistic autoantibody-mediated disease has been that of β_1_-adrenergic receptor autoantibodies observed in patients with dilated cardiomyopathy and in a rat model fulfilling Koch's postulates [5]. However, autoantibodies could also merely be an epiphenomenon in many instances; convincing data are still pending.

α_1_-adrenergic receptor (α_1_-AR) signaling mediates several cardiovascular actions such as vascular smooth muscle cell (VSMC) contraction, cardiac inotropy, hypertrophy, and remodeling [6]. α_1_-AR are predominantly located postsynaptically on VSMC, where they are the targets of circulating norepinephrine and regulate VSMC contraction [7]. Sympathetic over-activity in hypertension and accompanying excess stimulation of postsynaptic α_1_-AR supports the use of selective α_1_-AR inhibitors as antihypertensive drugs. However, α_1_-AR blockade was discontinued in the Antihypertensive and Lipid-Lowering Treatment to Prevent Heart Attack Trial (ALLHAT) because of a putative increased risk for heart failure [8], a highly controversial decision that has been sharply criticized [9]. The Valsartan Heart Failure Trial 2 (Val-HeFT2) showed that α_1_-AR blockade was beneficial in heart failure. α_1_-AR can contribute to cardiomyocyte hypertrophy. Huang et al demonstrated a protective effect of the α_1A_-AR-subtype in cardiac myocytes and defined an extracellular-regulated kinase (ERK) signaling pathway that is required for myocyte survival [10]. Hearts of α_1_-AR gene-deleted mice had increased interstitial fibrosis, increased apoptosis, and failed induction of the fetal hypertrophic gene program after pressure overload, indicating that α_1A_-AR are required for myocardial adaptation to stress [11], [12].

Others and we have described agonistic autoantibodies (α_1_-AAB) against the α_1_-AR [13], [14], [15]. Earlier, we examined immunoglobulin fractions in 54 severely hypertensive patients and found α_1_-AAB in 44% [15]. However, 12% of normotensive control subjects also harbored α_1_-AAB. Zhou et al. immunized rats with a peptide from the α_1_-AR epitope [16]. The rats developed α_1_-AR antibodies (α_1_-AB) and cardiac hypertrophy. However, the link between α_1_-AB formation and cardiac remodeling remains unclear. We have now performed additional studies to elucidate the pathophysiological relevance of α_1_-AAB. We show that removal of α_1_-AAB with immunoadsorption is feasible and lowers blood pressure. We raised α_1_-AB in rabbit and purified the α_1_-AB from rabbits and α_1_-AAB from patients by affinity chromatography. After confirming binding and functional specificity of the isolated rabbit α_1_-AB and human α_1_-AAB, we performed gene expression analysis in cardiomyocytes and vascular smooth muscle cells (VSMC). We show that both rabbit α_1_-AB and human α_1_-AAB can evoke calcium signaling and contract vessel preparations via protein kinase C alpha (PKC-α) activation and transient extracellular-related kinase (ERK 1/2) phosphorylation. Our data suggest that human α_1_-AAB can induce signaling pathways involved in hypertension and cardiac remodeling, suggesting a potential clinical relevance of α_1_-AAB.

## Results

We analyzed α_1_-AAB in 81 patients with refractory hypertension, who required ≥3 antihypertensive medications. Severe hypertension-induced target organ damage was present in every patient, including funduscopic changes, cardiac abnormalities, and kidney damage. Fourty-one patients (51%) featured α_1_-AAB as assessed by cardiomyocyte contraction assay. Microalbuminuria was detected in 33%, diastolic dysfunction in 87%, left ventricular (LV) hypertrophy in 85%, and reduced ejection fraction in 13% of α_1_-AAB positive patients. The patient characteristics are outlined in Table 1.

**Table 1:** Clinical features of the patients classified as α_1_-AR-AA positive or negative.

α_1_-AAB	Positive	Negative
Number	41	40
Age range	46–81 years	42–81 years
Male	59%	60%
Female	41%	40%
Medication classes	4.3	4.7
Goal blood pressure	77%	76%
Heart failure	64%	57%
Reduced ejection fraction	13%	24%
Diastolic dysfunction	87%	81%
Left ventricular hypertrophy	85%	76%
Microalbuminuria	33%	36%

**Figure 1:**
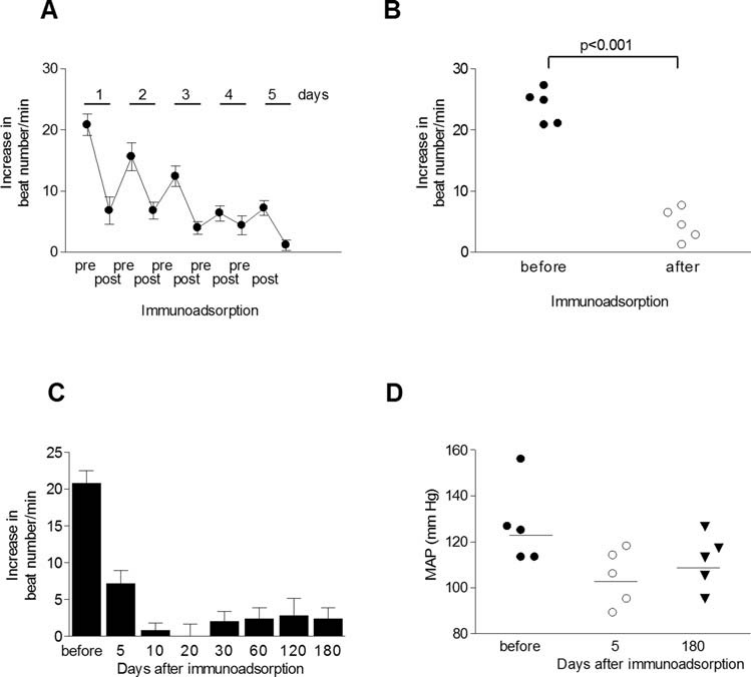
Effects of immunoadsorption on α_1_-AAB and blood pressure. (A) Ordinate shows neonatal cardiomyocyte spontaneous beating rate; abscissa shows response to immunoadorption performed on a representative patient. The α_1_-AAB activity decreased with every immunoadsorption with a total decrease in response over time. (B) Figure shows the initial spontaneous beating rate (closed circles). After five immunoadsorptions, this response is reduced to basal values (open circles) in all 5 patients. (C) Mean response of a representative patient in cardiomyocyte contraction assay over time to immunoadsorption is shown. (D) Mean arterial pressure (MAP) was measured 5 and 180 days after immunoadsorption. MAP was significantly reduced compared to before immunoadsorption.

Figure 1 shows data from the five α_1_-AAB positive patients undergoing immunoadsorption. The treatments were performed daily for 5 days. The cardiomyocyte contraction assay documented gradually decreasing α_1_-AAB activity during the course of the five immunoadsorptions (Figure 1A, B). At the end of five days, the activity was in the normal range. The decreased α_1_-AAB activity persisted for the 180 day observation period (Figure 1C). Blood pressure recordings performed before and after 5 days of treatment showed a significant reduction in mean arterial pressure (MAP), compared to initial values that remained significantly reduced for the observation period of 180 days (Figure 1D).

**Figure 2:**
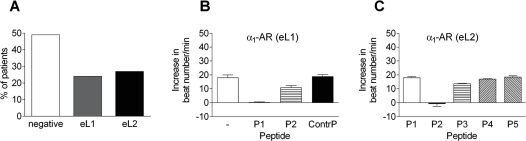
Epitope mapping for extracellular loop 1 and loop 2 of α_1Α_-AR is given. (A) α_1_-AAB were directed against the first extracellular loop (eL1) in 24% and against the second extracellular loop (eL2) in 27% of the hypertensive patients. (B) The peptide sequence P1 (YWAFGR) and P2 (GRVFCNI) (partially) for eL1 were able to diminish the spontaneous beating rate response completely. (C) Epitope mapping for loop 2 is given for the following amino acid sequences: P1: GWRQPA, P2: APEDET, P3: TICQIN, P4: INEEPG, P5: GYVLFS. The sequence P2 (APEDET) was able to diminish the spontaneous beating rate response completely.

α_1_-AAB were directed against the first extracellular loop (eL1) in 24% and against the second extracellular loop (eL2) in 27% of the hypertensive patients (Figure 2A). Epitope mapping for the first and second extracellular loop of the α_1A_-AR by cardiac contraction assay is shown in Figure 2B and C. Overlapping peptide sequences were tested for competition in cardiac contraction assay. For extracellular loop 1 (eL1) the peptide sequence P1 (YWAFGR) diminished the spontaneous beating rate response completely. The peptide sequence P2 (APEDET) competed for the antibody extracellular loop 2 (eL2) effect. Since activating autoantibodies against the second loop have already been described for the AT1-R and β_1_-AR receptor, we analyzed α_1_-AAB against the second extracellular loop of α_1A_-AR in this study.

**Figure 3:**
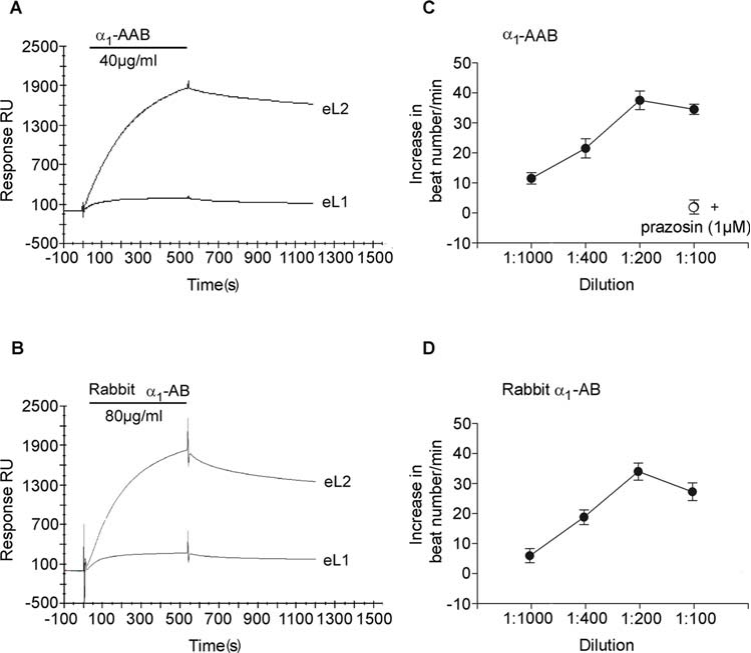
Binding and agonistic activity of patient α_1_-AAB and rabbit α_1_-AB. Surface plasmon resonance sensorgrams depicting antibody binding to α_1_-AR peptides (A and B). Biotinylated peptide corresponding to the α_1A_-AR extracellular loop2 (eL2) were immobilized. A corresponding peptide to extracellular loop1 (eL1) served as negative control. α_1_-AAB from a patient with refractory hypertension (A) and rabbit α_1_-AB (B) bind exclusively to eL2. Note that the characteristics of the human antibody (A) and rabbit antibody (B) were the same. (C) Functional effects (cardiomyocyte contraction assay) of affinity-purified α_1_-AAB (0.1–1 µg/ml medium) against second extracellular loop from a patient with refractory hypertension and (D) rabbit α_1_-AB (0.12–1.2 µg/ml medium) against second extracellular loop. The functional findings corroborate the BIAcor results.

We purified α_1_-AAB from patients by affinity chromatography using the peptide of the second extracellular loop of the α_1A_-AR. We then used surface plasmon resonance (SPR) measurements to determine the binding affinity and specificity of purified autoantibody fractions. Sensorgrams, depicted in Figure 3, demonstrate that the antibodies displayed a high binding affinity (Kd∼50 nM) to the biotinylated peptide corresponding to eL2 of the α_1_-AR immobilized to the SA-sensorchip. This affinity-purified antibody fraction also showed weak cross-reactivity with the biotinylated peptide corresponding to the eL1 of the α_1_-AR (Figure 3A). However, this binding was negligible compared to the strong eL2 interaction.

We next raised a peptide antibody against the second extracellular loop of the α_1_-AR (rabbit α_1_-AB) and purified the antibody on the same peptide-containing affinity matrix. In SPR experiments, the rabbit α_1_-AB produced regular association and dissociation kinetics reaching a signal of ∼2500 RU at a concentration of 80 µg/ml (Figure 3B). Purified α_1_-AAB from patients and rabbit α_1_-AB increased the beating rate of rat cardiomyocytes. This effect was dose-dependent (Figure 3C and D). The maximal response was obtained at an antibody dilution of 1∶200 (0.5 and 0.6 µg/ml medium, respectively). The positive chronotropic effect was blocked by the α_1_-AR antagonist prazosin (1 µM).

To elucidate the molecular pathways induced by α_1_-AAB, we performed gene expression studies. We used Affymetrix microarrays for quantification of mRNA expression in cardiomyocytes and VSMC after treatment with purified α_1_-AAB isolated from three different patients, rabbit α_1_-AB, human control IgG, and the α_1_-AR agonist phenylephrine (PE) for 24 h. We identified two genes with increased expression in the Affymetrix array that we then verified by TaqMan RT-PCR. These genes were *PLA2-IIA* and *Cacna1c,* as shown in Table 2. The upregulation of both genes was inhibited by the α_1_-AR antagonist prazosin while no changes in gene expression were observed after the treatment with the control IgG.

**Table 2:** Differential expression of *PLA2-IIA* and *Cacna1c* in cardiomyocytes and VSMC after treatment with patient α_1_-AAB, rabbit rabbit α_1_-AB or PE (Fold changes in TaqMan analysis).

	α_1_-AAB	rabbit α_1_-AB	PE
*PLA2-IIA*			
Cardiomyocytes	2.3	6.2	4.0
VSMC	4.8	2.5	5.1
*Cacna1c*			
Cardiomyocytes	2.2	1.7	4.0
VSMC	2.0	1.8	2.2

**Figure 4:**
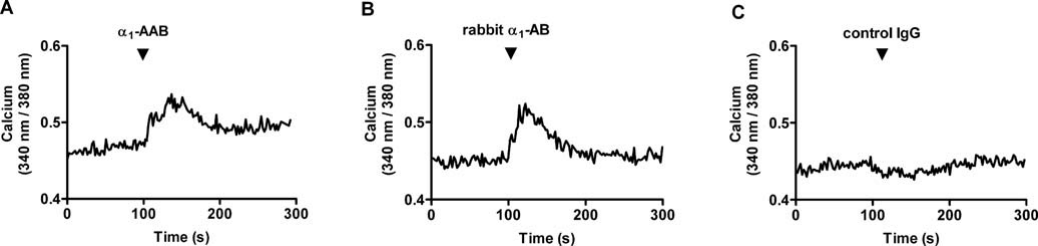
Representative traces of responses in intracellular Ca^2+^ of cultivated neonatal rat cardiomyocytes exposed to isolated patient α_1_-AAB and rabbit α_1_-AB are shown. (A) α_1_-AAB isolated from the serum of a patient with refractory hypertension elicited a Ca^2+^ signal. (B) The rabbit α_1_-AB gave a similar Ca^2+^ signal. (C) A human control IgG preparation was unable to affect intracellular Ca^2+^. Cardiomyocytes were electrically stimulated at 1 Hz, and the peak Ca^2+^ was monitored.

We tested whether or not α_1_-AAB from patients and rabbit α_1_-AB could elicit Ca^2+^ signals. Figure 4A shows the reaction of neonatal cardiomyocytes intracellular Ca^2+^ transients in response to the addition of the purified patient α_1_-AAB. There was a fast increase in the 340 nm/380 nm ratio, which peaked at less than 60 seconds and declined within 2 minutes to a plateau value slightly above the control level before the addition of the α_1_-AAB. Thus, α_1_-AAB potently induced a rise in intracellular Ca^2+^ in the targeted neonatal cardiomyocytes. To elucidate whether or not this Ca^2+^ response could be common to an autoantibody-α_1_-AR loop 2-type interaction, we used a rabbit α_1_-AB. As shown in Figure 4B, this rabbit α_1_-AB also generated an acute positive Ca^2+^ response in the cardiomyocytes with a similar time course as did the human antibody preparation. These responses were not elicited by a control IgG. As illustrated in Figure 4C, an IgG prepared from an α_1_-AAB negative patient was not able to raise the intracellular Ca^2+^.

**Figure 5:**
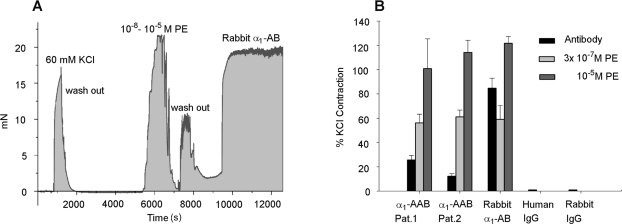
Contractile effect of patient α_1_-AAB and rabbit α_1_-AB in mesenteric arteries is given. A brisk KCl response is documented. (A) Representative experiment showing the contractile response to phenylephrine (PE, 10 nM–10 µM) and rabbit α_1_-AB (50 µg in 5 ml PBS buffer). The PE and antibody responses were similar in kind. (B) Contractile response of human α_1_-AAB isolated from two patients (5 µg in 5 ml PBS buffer) and rabbit α_1_-AB (50 µg in 5 ml PBS buffer) are compared to the PE response (300 nM and 10 µM, respectively) is shown. Contractions are expressed as % KCl response from patient 1 and patient 2. The human antibody responses were less than the low and high-dose PE responses. The rabbit α_1_-AB responses approached the PE responses. Two columns on the right are control human and control rabbit IgG showing no response.

These results prompted myographic experiments in mesenteric arteries displayed in Figure 5. Treatment with KCl documented brisk viability of the preparation. PE and the rabbit α_1_-AB both were able to constrict the vessel (Figure 5A). The effect of α_1_-AAB from two patients and from rabbit α_1_-AB was compared to half-maximal and maximal effective PE dosages, producing 50% and 100% of vessel constriction. Patient α_1_-AAB and rabbit α_1_-AB showed marked vasoconstriction activity. Control IgG from human and rabbits had no effect (Figure 5B).

**Figure 6:**
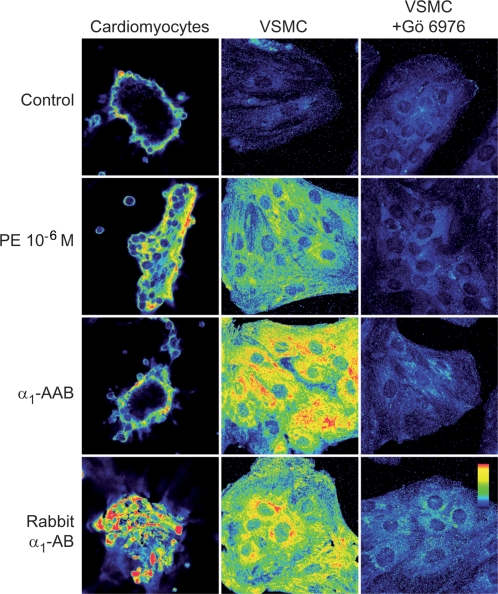
Protein kinase C alpha (PKC-α) activation in cardiomyocytes and vascular smooth muscle cells (VSMC) after 2 min incubation with PE, α_1_-AAB from patients, and rabbit α_1_-AB is demonstrated. Horizontal rows show regimens, namely, untreated control, PE, patient α_1_-AAB, and rabbit α_1_-AB. Vertical columns show cardiomyocyte and VSMC responses. Yellow shows PKC-α activation; red is more intense activation. Gö 6976 is a PKC-α inhibitor, which blocked all responses.

**Figure 7:**
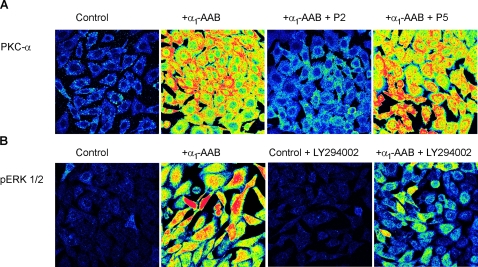
PKC-α and ERK 1/2 activation in CHO cells stably transfected with human α_1Α_-AR (CHO/α_1Α_-AR) by α_1_-AAB is demonstrated. (A) Incubation of CHO/α_1Α_-AR cells with α_1_-AAB for 2 min resulted in a PKC-α activation, which was blocked by the peptide P2, but not by the peptide P5. (B) ERK 1/2 phosphorylation after incubation with α_1_-AAB for 5 min is shown. Inhibition of the upstream phosphatidylinositol 3 (PI3)-kinase activity by the inhibitor LY294002 strongly reduced ERK 1/2 phosphorylation. Yellow shows PKC-α and ERK 1/2 activation, respectively; red is more intense activation.

We then examined signal transduction. We concentrated on PKC-α and ERK 1/2 as both have been shown to be important in α_1_-AR-stimulated hypertension-induced target organ damage [6]. As shown in Figure 6, we found that α_1_-AAB and rabbit α_1_-AB exposure to cardiomyocytes or VSMC resulted in PKC-α activation, as did the positive PE control. These effects were blocked completely by the specific PKC-α blocker, Gö 6976. Additionally, the incubation of Chinese hamster ovary (CHO) cells stably transfected with human α_1Α_-AR (CHO/α_1Α_-AR) with α_1_-AAB resulted in PKC-α activation, which was blocked by the peptide P2, corresponding to the binding site of the α_1_-AAB (Figure 7A).

**Figure 8:**
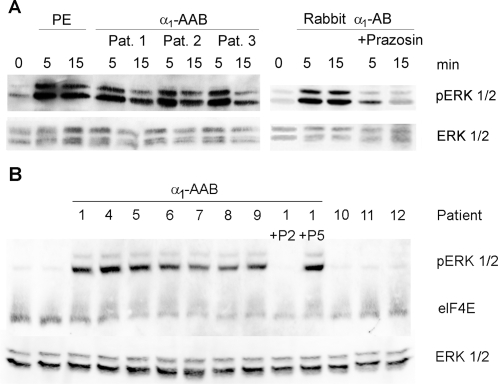
Activation of ERK 1/2 in response to treatment with PE, α_1_-AAB from patients or rabbit α_1_-AB is shown. (A) Cardiomyocytes were treated for 5 and 15 min with 10 µM PE, 2.5 µg/ml α_1_-AAB or rabbit α_1_-AB, respectively. Equivalent amounts of protein were analyzed by Western blotting with anti-pERK 1/2 antibody (44/42 kD) and the amount of ERK was analyzed with anti-ERK 1/2 antibody. PE, patient α_1_-AAB, and rabbit α_1_-AB caused ERK 1/2 phosphorylation. (B) CHO/α_1Α_-AR cells were incubated with patient α_1_-AAB for 5 min. Lane 1 and 2 represent untreated cells. Patients 1, 4–8 were α_1_-AAB-positive in the cardiomyocyte contraction assay. “Patient” 9 represents a pool of five α_1_-AAB positive patients. Patients 10–12 were α_1_-AAB negative in the cardiomyocyte contraction assay. Their serum samples were processed identically to those of α_1_-AAB-positive patients. ERK 1/2 phosphorylation was inhibited by peptide P2, but not by peptide P5. Eukaryotic initiation factor 4E (elF4E, 25 kD) antibody was used as translational control, the amount of ERK was analyzed with anti-ERK 1/2 antibody.

We then studied ERK 1/2 phosphorylation and the upstream phosphatidylinositol 3 (PI3)-kinase activity in CHO/α_1Α_-AR cells. Incubation with human α_1_-AAB resulted in ERK 1/2 phosphorylation. The PI3-kinase inhibitor, LY294002, inhibited the ERK 1/2 phosphorylation (Figure 7B). Additional hypertensive patients, who were either α_1_-AAB positive or negative in cardiomyocyte contraction assay, were characterized by ERK 1/2 activation with immunoblotting (Figure 8). α_1_-AAB from three patients induce a transient ERK 1/2 phosphorylation similar to that evoked by PE and rabbit α_1_-AB in neonatal cardiomyocytes. Phosphorylation was blocked by prazosin (Figure 8A). A similar activation was obtained in CHO/α_1Α_-AR cells. The activation was specifically blocked by peptide P2 (Figure 8B). Fractions eluted from patients, who were negative in the cardiomyocyte contraction assay, failed to activate ERK 1/2.

## Discussion

The important findings in this study are that isolated α_1_-AAB und generated α_1_-AB show similar binding characteristics by surface plasmon resonance sensorgram and functional testing. The antibodies induce a Ca^2+^ dependent signal transduction cascade in VSMC and cardiomyocytes that are both important to the pathophysiology of hypertension and cardiac remodeling. By gene array studies, we showed that the isolated patient α_1_-AAB and rabbit α_1_-AB both upregulate two gene types, namely *PLA2-IIA* and *Cacna1c*. In a proof-of-concept, non-controlled trial, we show that removal of α_1_-AAB by immunoadsorption lowers blood pressure in α_1_-AAB positive patients with intractable primary hypertension.

The entire idea of “agonistic” autoantibodies contributing to cardiovascular disease is controversial [17]. Antibodies directed at receptors can block or stimulate their targets. Hallmark example of the latter action is Graves' disease where antibodies directed at the thyroid stimulating hormone receptor exert an agonistic action. Recently, compelling evidence has been presented regarding agonistic antibodies directed against the α_1_-AR, the β-ARs, the Ang II AT1-R, and the platelet-derived growth factor-alpha receptor, as reviewed elsewhere [17]. The antibodies could play a pathogenic role in various cardiovascular diseases, including hypertension, cardiomyopathy, preeclampsia, acute humoral rejection, and connective tissue disease.

Earlier, we speculated that α_1_-AAB could contribute to hypertension [15]. Our goal here was to give the notion of α_1_-AAB a more robust scientific basis. Thus, we verified our candidate by generating α_1_-AB by immunization. Concomitantly, we subjected patients from our collective who harbored α_1_-AAB to immunoadsorption. We found in our uncontrolled trial that lowering α_1_-AAB titers was associated with a lower blood pressure. We did not perform a randomized double blind, crossover trial. Nonetheless, our study meets class I (safety) criteria and suggests that a future class II (efficacy) trial might be warranted. We cannot exclude the possibility that short-term effects of the immunoadsorption, such as circulating blood volume changes during apheresis or other confounders contributed to the reduction in blood pressure. Since the reduction in blood pressure persisted for the observation period of 180 days, we believe that removal of α_1_-AAB by apheresis was the main cause for the blood pressure reduction. Within the five days immunoadsorption period, the level of α_1_-AAB decreased gradually. Thereafter, α_1_-AAB could not be detected after successful immuoadsorption in these patients for the entire observation period of 180 days. A similar state-of-affairs has been described for β_1_-AAB in dilated cardiomyopathy after immunoadsorption. These data suggest that immunomodulation by removal or neutralization of the AAB might represent a potential therapeutic strategy [18].

We isolated the detected α_1_-AAB and the α_1_-AB that we generated against the same epitope in rabbits, using the same affinity purification protocol. Since we used the same peptide sequence for epitope mapping in the cardiomyocyte contraction assay, immunization in the rabbit, and isolation in the affinity column, we are confident that we isolated solely α_1_-AB. Before using this material to learn more about α_1_-AR signaling in VSMC and cardiomyocytes, we confirmed binding specificity by functional (cardiac contraction assay and ERK 1/2 phosphorylation) and biochemical (surface plasmon resonance measurement) verification methods. We then performed an Affymetrix gene expression study. We followed up on the results to pursue two candidate genes, *PLA2-IIA* and *Cacna1c* that were upregulated in the display, as verified by RT-PCR. Both genes contribute to signaling pathways in hypertension and atherosclerosis. Phospholipases A2 are acute-phase reactants and play an important role in digestion and metabolism of phospholipids, as well as in production of precursors for inflammatory reactions. Plasma PLA2-IIA levels are increased in systemic inflammation, including, rheumatoid arthritis and cardiovascular diseases [19], [20], [21]. In infarcted hearts, expression of PLA2-IIA was markedly increased in damaged cardiomyocytes [22]. Inhibition of PLA2-IIA also prevented cardiac fibrosis in spontaneously hypertensive rats [23].

Another important finding accrued is the fact that both the α_1_-AAB from patients and the rabbit α_1_-AB affected intracellular Ca^2+^ at two different levels, namely the acute, short-term elevation of intracellular Ca^2+^, and the increased transcript expression of the voltage-gated L-type Ca^2+^ channel pore subunit. Acute administration of the purified antibodies to neonatal cardiomyocytes produced a typically shaped Ca^2+^ transient. The onset of the cytosolic Ca^2+^ response occurred within few seconds reaching its maximum at less than one minute. α_1_-AR stimulation potentiates L-type Ca^2+^ current through CaMK II activation in rat ventricular myocytes [24]. Furthermore, rabbit antibody to the α_1_AR and autoantibodies against the AT1-receptor could activate the Ca^2+^ current [25], [26]. The peak response of cytosolic Ca^2+^ to the antibodies apparently comprises a temporary imbalance of Ca^2+^ entry through L-type Ca^2+^ channel and the sarcoplasmic reticulum Ca^2+^ release on one hand and Ca^2+^ sequestration into the sarcoplasmic reticulum and Ca^2+^ extrusion via the Na^+^/Ca^2+^ exchanger on the other hand.

In addition to acute Ca^2+^ current stimulation, we found that long-term activation of the α_1_-AR pathway by patient α_1_-AAB and rabbit α_1_-AB increases transcript levels of the voltage-dependent L-type Ca^2+^ channel α1C subunit (*Cacna1c*) in neonatal cardiomyocytes and VSMC. Voltage-dependent L-type Ca^2+^ channels (L-VDCC) play a crucial role in the regulation of heart rhythm and contractile performance, as well as in smooth muscle tone. The channels open in response to membrane depolarization providing Ca^2+^ ingress into the cell. In the myocardium, this local rise in Ca^2+^ concentration triggers massive Ca^2+^ release from the sarcoplasmic reticulum stores inducing in turn contraction. L-VDCC expressed in cardiac and smooth muscle are multi-subunit proteins containing the α1C, α2/δ, and β subunits. The α1C subunit forms the ion-selective pore, determines voltage sensitivity, and is a binding site for clinically used Ca^2+^ channel blockers [27]. Thus, upregulation of α1C subunit expression in response to α_1_-AR stimulation observed herein provides the route for an enhanced Ca^2+^ ingress which may contribute to Ca^2+^ imbalances and translate in cardiac dysfunction. Our results are consistent with those of Pignier *et al*. who reported on increased expression of functional L-type Ca^2+^ channels and hypertrophy in neonatal cardiomyocytes upon chronic α_1_-AR stimulation [28]. In contrast, Maki *et al*. described that in neonatal rat cardiomyocytes activation of the α_1_-AR pathway was accompanied by attenuated α1C mRNA levels [29]. A link between increased α1C protein levels and hypertrophy has also been demonstrated for the human heart [30].

A transgenic mouse with cardiac specific over-expression of solely the Ca^2+^ channel pore subunit has been studied intensively [31]. When α1C protein levels were increased by about 3-fold, a slightly increased Ca^2+^ inward current (30–40%) was observed, and the mice developed hypertrophy and severe cardiomyopathy as a function of age. The small sustained ingress of Ca^2+^ through the channel is capable of elevating PKC-α before the development of hypertrophy. [32] Activation of PKC in adult cardiomyocytes regulates ERK 1/2 through the c-RAF/MEK/ERK signaling pathway and α_1_-AR-stimulated hypertrophy in cardiomyocytes is mediated via activation of the Ras-Raf-MEK1/2-ERK 1/2 signaling pathway [6], [33]. As a consequence, we investigated the regulation of PKC-α and ERK 1/2 activity by human α_1_-AAB, rabbit α_1_-AB, or PE. We demonstrated that α_1_-AAB and α_1_-AB induced PKC-α stimulation and ERK 1/2 phosphorylation. Finally, we showed that human α_1_-AAB and the rabbit α_1_-AB evoke vasoconstriction in a vascular bed.

Taken together, we present data from a limited, uncontrolled, human trail of α_1_-AAB removal from severely hypertensive human subjects. This trial presents a hypothesis-generating result. We show that isolated α_1_-AAB from man and rabbits cause gene upregulation important to hypertension-relevant pathways and then document the induction of Ca^2+^-dependent pathways. We provide evidence that α_1_-AAB are of potential pathophysiological relevance and could represent a factor contributing to the development of refractory hypertension. Removal or neutralization of α_1_-AAB might represent a possible therapeutic option. Accordingly, we suggest that the entire gamut of autoimmunity in chronic cardiovascular disease warrants further critical inspection.

## Materials and Methods

### Patients

Patients requiring three or more medication classes to achieve goal control blood pressure values were recruited from the Franz-Volhard Clinic outpatient department. All had been studied intensively and secondary causes of hypertension had been ruled out. Home and clinic blood pressures in these patients were determined with an automated oscillometric device under standardized conditions. The internal review board approved the study and written informed consent was obtained from all participants. Five patients harboring α_1_-AAB were asked to undergo five immunoadsorption treatments with an especially prepared column according to procedures described elsewhere [34].

### α_1_-AAB detection

We isolated immunoglobulin fractions from serum samples as described earlier [4], [15]. For detection of autoantibodies, the immunoglobulin fractions were added to neonatal rat cardiomyocytes at a dilution of 1∶20. For the neutralization experiments, synthetic peptides corresponding to the sequence of the first extracellular loop (YWAFGRVFCNIWA), and the second extracellular loop (PAPEDETICQINEE) of the human α_1Α_-AR were each added in excess (0.05 to 0.1 µg) to the immunoglobulin fraction. The mixtures were shaken and placed in a refrigerator for 1 h. The 100 µl samples were then added to neonatal rat heart muscle cells cultured in 2 ml of medium to a final dilution of 1∶40. The beating rate was counted for 15 sec, 5 and 60 min after the addition of the peptide/immunoglobulin mixture. We tested α_1_-AAB activity in a neonatal cardiomyocyte assay without and with peptide inhibition. Pulsation rate was exhibited 1 h or 24 h, respectively after incubation with α_1_-AAB and compared with the spontaneous basal pulsation rate. Eight synchronously contracting cell clusters per flask were counted for 15 sec on a heat table stage.

### α_1_-AB generation in rabbit and antibody purification

We immunized rabbits against a peptide corresponding to the amino acid sequence of the second extracellular loop of the isoform A of human α_1_-AR (PAPEDETICQINEE) by BioGenes GmbH (Berlin, Germany). The corresponding peptide of the second extracellular loop of the α_1_-AR was covalently bound to ε-aminocapryl agarose (Sigma-Aldrich, Munich, Germany) to yield epitope-specific affinity beads. For coupling the agarose gel matrix (∼1 ml of packed gel) was activated by glutaraldehyde (1% solution, freshly prepared) for 20 min at room temperature on a rotating wheel. The activated gel was extensively washed with PBS and allowed to react with the peptide solution for 3 h at room temperature or overnight at 4°C on a rotating wheel. The coupling reaction was terminated by incubation with 200 mM Tris/Glycine buffer pH 7.2. The affinity beads were poured into a column (Bio-Rad, Munich, Germany) and stored with 0.02% sodium acid in buffer A consisting of 50 mM Tris/HCl, 0.5 M NaCl, pH 7.4, at 4°C.

The affinity beads were washed with buffer A to remove the sodium acid. Serum samples (5–15 ml) from patients with refractory hypertension or from immunized rabbits were incubated with the affinity beads overnight at 4°C on a rotating wheel. The beads were allowed to settle and the sera were removed and stored for a second round of affinity purification at a given matrix. The beads were washed with buffer A until baseline levels of protein are detected at 280 nm. The antibodies were eluted at room temperature in 1 ml fractions with 50 mM Tris/Glycine, 0.5 M NaCl, pH 2.5. Antibody fractions were immediately neutralized with 0.5 ml of 0.5 M Tris/HCl, 0.5 M NaCl, pH 7.4. The antibody concentration was calculated by measurement the absorption at 280 nm.

### Surface plasmon resonance measurements

Binding experiments were performed in a BIAcore 2000 Instrument (Uppsala Sweden) at 25°C. N-terminally biotinylated peptides corresponding to the first and second extracellular loop of the α_1A_-AR were immobilized at binding levels of 100 relative units (RU) each on parallel lanes of a SA-biosensor chip. Affinity-purified antibodies from patient blood samples were injected in the flow cells at a rate of 20 µl/min in HBSE running buffer consisting of 10 mM HEPES, pH 7.4, 150 mM NaCl, and 3 mM EDTA. The binding was regenerated between binding measurements using 5 mM Tris/Glycine, 50 mM NaCl, pH 2.5, with no decrease in extent measurements over the duration of an experiment. Data were analyzed using the using the BIAevaluation 3.2 RC 1 program. The analysis software corrects for baseline drift during measurements. The curves were fitted to a single-site interaction model. Kd values were calculated by using the formula Kd = k*_off_* /k*_on_* in which k*_off_* and k*_on_* is the rate constant of dissociation and association kinetics, respectively.

### Cell culture and autoantibody incubation

Rat neonatal cardiomyocytes were prepared from ventricles of 1–2 day-old Wistar rats using a modified method [35]. The cells were cultured as monolayers for 4 days at 37°C in SM 20-1 medium supplemented with 10% heat-inactivated calf serum, 2 µM fluorodeoxyuridine and penicillin/streptomycin. Aortic VSMC were isolated from Sprague Dawley rats as described previously [36]. CHO cells were stably transfected with human α_1Α_-AR (CHO/α_1Α_-AR) using a pSW104 vector and were cultured in F12 HAM medium supplemented with glutamine, 10% FCS and 1% penicillin/streptomycin as described earlier [37]. For gene expression analysis cardiomyocytes and VSMC, respectively were incubated with human control IgG endobulin (5 µg/ml medium, Baxter, Wien, Austria), α_1_-AAB from different patients (2.5 µg/ml medium), rabbit α_1_-AB (2.5 µg/ml medium), and with the α_1_-AR agonist PE (10 µM, Sigma-Aldrich) for 24 h in DMEM medium containing 1% serum. Experiments were repeated with three different cardiomyocytes and VSMC preparations. For investigation of protein phosphorylation, cardiomyocytes and CHO/α_1Α_-AR cells were maintained in serum-free media for 24 h or 4 h, respectively and treated with PE, human α_1_-AAB or rabbit α_1_-AB for 5 and 15 min, respectively. For inhibition experiments, prazosin (1 µM, Sigma-Aldrich) was added. Five µg of the peptides P2 (APEDET) or P5 (GYVLFS) were given to 2.5 µg of α_1_-AAB 1 h before cell treatment. For the inhibition of ERK 1/2 activation, CHO/α_1Α_-AR cells were pre-incubated with PI3-kinase inhibitor LY294002 for 10 min.

### Gene expression analysis

We extracted total RNA from cardiomyocytes treated with human control IgG, α_1_-AAB, rabbit α_1_-AB or PE using the RNeasy Purification Kit (Qiagen GmbH, Hilden, Germany). RNA was treated by deoxyribonuclease I (Qiagen). Two µg RNA of cells were transcribed in cRNA with One-Cycle Target labeling and Control Reagents (Affymetrix, Santa Clara, CA, USA). Non-pooled microarray experiments were performed with cRNA prepared from independent cardiomyocyte cell preparations using Rat Genome 230 2.0 Arrays (31,099 probe sets, Affymetrix). After passing the quality control for each experiment a set of RMA normalized expression values have been produced. The log scale robust multi-array analysis (RMA) estimates are based upon a robust average of log2 (B (PM)), where B (PM) are background corrected perfect match intensities [38]. For statistical comparison of expression data student's t-test was used.

### Quantitative Real-Time Reverse Transcriptase PCR (TaqMan)

cDNA was synthesized from 2 µg of total RNA isolated from cardiomyocytes and VSMC, respectively using PowerScript Reverse Transcriptase (BD Bioscience Clontech, Palo Alto, USA) and an Oligo (dT)_18_ primer. Real-time PCR experiments were done using the Mx3000P® real-time PCR system (Stratagene Europe, Amsterdam, NL) and the Brillant QPCR master mix (Stratagene Europe). Real-time PCR was performed with non-pooled samples from cardiomyocytes and VSMC respectively. Beta-2-microglobulin (*B2M*) and glyceraldehyde-3-phosphate dehydrogenase (*GAPDH*) were used as endogenous references to normalize expression of a target gene. For every sample three independent runs in triplicates and the relative changes in gene expression were quantified by comparative Ct method [39]. Primer sequences are listed in Table 3.

**Table 3:** Primer and probe sequences used for TaqMan RT-PCR.

Gene		Sequences (5′→3′)	Accession
B2M	for	GCT CGG TGA CCG TGA TCT TT	NM_012512
	rev	GAG TTT TCT GAA TGG CAA GCA	
	probe	FAM-TGG TGC TTG TCT CTC TGG CCG TC-TAMRA	
GAPDH	for	CAA CGG CAC AGT CAA G	NM_017008
	rev	TCG CTC CTG GAA GAT G	
	probe	FAM-TGA GAA TGG GAA GCT GGT CAT CA-TAMRA	
PLA2-IIA	for	GGA CTC CTG CCG GAA ACA G	NM_031598
	rev	TTC CGG GCA AAA CAT TCA G	
	probe	FAM-TGT GCC AGT GCG ATA AAG CTG CC-TAMRA	
Cacna1c	for	TCA CTG CTG TCG GGA TAA GC	NM_012517
	rev	GGC CTT CTC CCC TGA AAA G	
	probe	FAM-AGC TGG GCG GTG TAC GAA GTC G-TAMRA	

Used annealing temperature was 58°C.

### Immunocytochemistry

We described the techniques for confocal microscopy and immunocytochemistry as referenced previously [4], [40]. The cells were fixed with 4% paraformaldehyde and permeabilized with 80% methanol at −20°C. After incubation with 2% BSA in PBS for 60 minutes, the preparation was incubated for 1 hour at room temperature with the monoclonal anti-PKC-α antibody from UBI (clone M4) diluted in PBS with 1% BSA (1∶80) or pERK 1/2 antibody (1∶200, Cell Signaling Technology, Boston, MA, USA), respectively, washed twice with PBS, and then exposed to the secondary antibody (Alexa-488-conjugated anti-mouse IgG at 1∶200, 1% BSA/PBS; Invitrogen, Life Technologies, Carlsbad, CA, USA) for 60 minutes. The preparation was mounted with AquaPolymount (Polyscience, Niles, IL, USA) under a glass coverslip on a Nikon-Diaphot microscope. An MRC 1024 confocal imaging system (Bio-Rad) with an argon/krypton laser was used. At least 25 to 40 cells from each of at least three experiments were examined under each experimental condition.

### Western blotting

Cardiomyocytes and CHO/α_1Α_-AR cells were scraped in lysis buffer [20 mM HEPES (pH7.9), 350 mM NaCl, 20% glycerol, 1 mM MgCl2, 0.5 mM EDTA, 0.1 mM EGTA, 1% NP40, complete protease inhibitor cocktail (Roche Diagnostic GmbH, Mannheim, Germany) and phosphatase inhibitor cocktail (Sigma-Aldrich)] and centrifuged at 10,000 rpm for 10 min. Protein concentrations were measured with Bradford method and equal amounts of proteins were analyzed by Western blotting. ERK phosphorylation was detected by pERK 1/2 antibody (1∶1000, Cell Signaling Technology). As translational control an ERK antibody (1∶1000, Cell Signaling Technology) and eukaryotic initiation factor 4E (elF4E) antibody (1∶7000, Cell Signaling Technology) were used. HRP-conjugated goat anti-rabbit secondary antibody (1∶5000, Jackson Immunoresearch Europe, Suffolk, UK) was used. Detection was performed with the ECL™-substrate (Lumigen, Southfield, MI, USA) according to the manufactureŕs instructions.

### Measurement of cytosolic Ca^2+^ transients

Neonatal cardiomyocytes were plated onto Labtek four chamber slides (Nunc GmbH & Co, Wiesbaden, Germany) suited for fluorescence measurements at a densitiy of 0.2×10^6^ cells per chamber. After cultivation for four days the medium was removed and cells were washed twice with 10 mM Hepes-buffered Hank's salt solution, pH 7.4 (HBSS). Cardiomyocytes were incubated on HBSS for 60 minutes at 37°C. Then the solution was replaced by 0.5 ml HBSS containing 2.5 µM final concentration of Fura 2-AM (Calbiochem AG, Luzern, Switzerland) and left for loading in the dark at room temperature. After 30 min the loading solution was aspirated, the cells washed and kept on 0.5 ml HBSS in the dark and at room for another 30 minutes before use. Measurements of cytosolic Ca^2+^ transients were performed on an IonOptix Fluorescence and Contractility System (Milton, MA, USA) equipped with a Leica microscope with a heatable stage. All measurements were carried out at 37°C. Cardiomyocytes were electrically stimulated at 1 Hz and the ratio of 340 nm/380 nm was recorded. After stabilization of the Fura signal the control trace was taken. Antibody preparations were applied in a constant volume of 100 µl of pre-warmed HBSS to give a final concentration of 2 µg/ml. For evaluation of Ca^2+^ changes peak values were derived from the trace recordings representing the maximum cytosolic Ca^2+^ achieved in the contracted cell.

### Contraction of mesenteric arteries

Vessel rings from superior mesenteric arteries of male Sprague-Dawley rats (200 to 300 g, 6 to 8 weeks) were prepared and intracellular membrane potential was measured as earlier described [41]. In the first series of experiments, the rings were exposed to increasing doses of PE (10 nM–10 µM) and with acetylcholine (10 nM–10 µM) for relaxation. Following, vessel rings were incubated with α_1_-AAB (5 µg in 5 ml PBS buffer), rabbit α_1_-AB (50 µg in 5 ml PBS buffer), rabbit IgG (50 µg in 5 ml PBS buffer, Dunn Labortechnik GmBH, Asbach, Germany) and human control IgG endobulin (50 µg in 5 ml PBS buffer).

### Statistics

We relied on student's t-tests (adjusted as necessary) for normally distributed data and performed analysis of variance (repeated measures were indicated). P<0.05 was accepted as significant. Data are expressed as mean±SD.
